# Bibliographical Notices

**Published:** 1854-07

**Authors:** 


### EDITORIAL DEPARTMENT.

### BIBLIOGRAPHICAL NOTICES.

*Chemical Lectures on Pulmonary Consumption.* By THEOPHILUS THOMPSON, M. D., F. R. S., &c. Philadelphia. Lindsay & Blakiston, 1854, pp. 259.

FEW diseases have been more carefully studied than the melancholy one of which these lectures treat. Preeminently the scourge of the gifted, the beautiful, the refined, it is invested with a fearful charm to all who approach it. Then the sad conclusion to which the physician, who has studied every fibre of the frame, every circumstance of the disease, must come, the futility of attempting any thing beyond securing an euthanasia, adds to the melancholy interest of the whole subject.

Much, however, as this affliction has been studied, the complaints have been numerous, and not altogether unjust, that the method of examination has become more and more objectionable. Too much attention has been bestowed upon the physical signs arrived at by mere stethoscopic examination. The minds of pupils, especially, have been entirely too much concentrated upon this method of study. This is objectionable, as all one-sidedness in medicine is objectionable. It prevents that expansive and all-comprehending survey of the phenomena of disease, without which medical philosophy is impossible.

The volume before us appears to us a return to a better method. Without neglecting auscultation and percussion, which none but a deaf man or a grossly incompetent one could think of, the author does not force them into that exclusive preeminence to which their friends consider them entitled. He regards them as symptoms only, to be taken in connection with other symptoms, in order to arrive at a true idea of the disease.

The author has enjoyed excellent opportunities for the study of this very important disease, being the physician to the Hampton hospital for diseases of the chest. The lectures took each its specific color and direction, from the circumstances of the cases which were examined at each. In consequence of this, we have interesting disquisitions on a variety of individual topics, each one of which is of importance to the practitioner of medicine. The pulse, the expectoration, the chemistry of the blood, the conditions of the urine, are all separately considered and each is treated in a plain, practical manner, which cannot fail to be acceptable to the practicing physician.

We were glad to see that Mr. Thompson speaks favorably of the action of cod liver oil in consumption. He narrates some cases which are much stronger than any we were prepared to expect. The healing of vomicæ as exhibited by the physical signs, at the same time that the weight increased and the strength improved, is certainly a fact calculated to inspire the physician with renewed hope for the patients suffering under this deadly blight.

We strongly recommend the book to both student and practitioner. There is nothing in it, from title page to finis which is not eminently practical.

---

*The Science and Art of Surgery,* being a Treatise on Surgical Injuries, Diseases and Operations. By John Erichsen, Professor of Surgery in University College and Surgeon to University Hospital. Edited by John H. Brinton, M. D. Illustrated by 311 engravings on wood. Philadelphia. Blanchard & Lea, 1854, pp. 908.

The motto of this book is a suggestive remark of the great Chancellor: "They be the best chirurgeons which, being learned, incline to the traditions of experience, or being empirics incline to the methods of learning." It is the production of a man who is at once a philosopher and practitioner, a careful observer of facts, and an assiduous student of other men's observations.

To pretend to give any idea of such a work in the short limits of a bibliographical notice would be absurd. We shall only say that it is a full and candid statement of the present condition of surgical science and art.

The arrangement of the book is somewhat peculiar, as must necessarily be the case when an author thinks for himself. Every man must arrange his subject in that order in which it is most lucid to his own mind, and when there is no idiosyncracy manifest in the arrangement, there will usually be found no individuality in the thoughts of a book.

The author begins with an account of congestion, determination and inflammation. These important subjects are amply elucidated by numerous references to physiological experiments and engravings of microscopical phenomena. Then follows an account of the general principles of operation, particulary of amputations. These subjects comprise one division of the book, called by the author first principles.

The second division treats of surgical injuries. These include the general effects of injury, such as shock, traumatic delirium, &c. Injuries of the soft parts in general are then examined, the various kinds of wounds, &c., after which follow wounds of nerves, veins and arteries, the latter class being divided into wounds of these vessels generally, and wounds of special arteries. Injuries of muscles and tendons, of bones, of joints, of the cranium, the spine, the face, &c., and the whole division is closed by burns, scalds and frost-bite.

The third and last division comprehends surgical diseases. Abscess, ulcers, mortification, erysipelas, pyemia, tumors, scrofula and syphilis, as general diseases are first considered. Then diseases of special structures are taken up, and lastly, diseases of particular organs are considered.

The reader will perceive that a rigid and exact system pervades every department of the book, and we need only add that the plan is as well executed as conceived. The cuts are numerous and good and the whole book very well gotten up.

---

*Turkey and the Turks.* By Dr. J. V. C. Smith, author of a pilgrimage to Palestine, &c. Boston. James French & Co., 1850, pp. 320.

At the present time when the attention of the whole civilized world is directed to the east, people naturally desire the opinion of some competent and well informed observer, in regard to the state of the contending parties and the probable issue of the quarrel. As far as Turkey is concerned, the present condition of that country can be satisfactorily ascertained from the book before us. Dr. Smith spent some time in the Ottoman empire, and gives a very interesting sketch of the habits, manners and political condition of the Turks. He is a close observer and an agreeable writer.

---

*The American Journal of Science and Arts,* for July, comes to us as usual, stored with valuable matter.

Contents: on the first hurricane of September, 1853, by W. H. Redfield; account of a rainbow caused by light reflected from water, by E. S. Snell; a change of sea level effected by existing physical causes during stated periods of time, by Alfred Tyler; on the phosphate of iron and manganese from Norwich, by J. W. Mallet; on the homœomorphism of mineral species of the trimetric system, by J. D. Dana; on certain physical properties of light, produced by the combustion of different metals, in the electric spark, by David Alter; Chinese and Aztec Plumagery, by D. J. MacGowan; Binocular microscope, by E. D. North; mechanical action of heat, by W. J. M. Rankin; the Brandon tornado of January 20th, 1854, by E. Loomis; considerations on the group of small planets situated between Mars and Jupiter, by Le Verrier; on electric induction, by Faraday; reclamation of borocalcite as distinct from a mixture of minerals, by A. A. Hayes; on the present condition of the crater of Kilauea, Hawaii, by T. Coan; note on the genus Buckleya, by Asa Gray; on the mode of giving permanent flexibility to brittle specimens in zoology and botany, by J. W. Baily; caricography, by C. Dewey; reviews and records in anatomy and physiology, by W. J. Burnett; correspondence of J. Nicklés; scientific intelligence; lists of new publications.

We have already expressed our opinion so freely of the merits of this periodical, that it can scarcely be necessary for us to add any thing more. We will, however, say that we consider it essential to every man who wishes to keep up with the progress of general science in this country.

*An Address to the Public in Regard to the Affairs of the Medical Department of Hampden Sydney College.* By several physicans of the city of Richmond. Richmond, 1853.

OUR readers generally are acquainted with the fact that there has been for several years past, a successful medical school in operation in the city of Richmond. Being a branch of Hampden Sydney College, it has no charter, of its own, but acts under that of the parent institution. This fact has recently given rise to a breach between the faculty of the medical department and the president and trustees of the college. The faculty desire the filling up of vacancies which may occur in their body, while the trustees refuse to grant them any such permission, reserving it to themselves as one of their original and inalienable rights.

The present pamphlet is a vindication of the claims of the trustees, and contains the legal opinion of Messrs. Watson and Hughes, fully sustaining the views of the trustees.

